# The Natural Fungal Metabolite Beauvericin Exerts Anticancer Activity In Vivo: A Pre-Clinical Pilot Study

**DOI:** 10.3390/toxins9090258

**Published:** 2017-08-24

**Authors:** Daniela Heilos, Yelko Rodríguez-Carrasco, Bernhard Englinger, Gerald Timelthaler, Sushilla van Schoonhoven, Michael Sulyok, Simon Boecker, Roderich D. Süssmuth, Petra Heffeter, Rosa Lemmens-Gruber, Rita Dornetshuber-Fleiss, Walter Berger

**Affiliations:** 1 Institute of Cancer Research, Department of Medicine I, Medical University of Vienna, and Comprehensive Cancer Center of the Medical University, Borschkegasse 8a, 1090 Vienna, Austria; daniela.heilos@univie.ac.at (D.H.); bernhard.englinger@meduniwien.ac.at (B.E.); gerald.timelthaler@meduniwien.ac.at (G.T.); sushilla.vanschoonhoven@meduniwien.ac.at (S.v.S.); petra.heffeter@meduniwien.ac.at (P.H.); rita.dornetshuber@univie.ac.at (R.D.-F.); 2 Department of Pharmacology and Toxicology, University of Vienna, Althanstr. 14, 1090 Vienna, Austria; rosa.lemmens@univie.ac.at; 3 Department of Food Chemistry and Toxicology, Faculty of Pharmacy, University of Valencia, Av. Vicent A. Estellés s/n, 46100 Burjassot, Spain; yelko.rodriguez@uv.es; 4 Department of Agrobiotechnology (IFA-Tulln), University of Natural Resources and Life Sciences, Vienna (BOKU), Konrad Lorenz Str. 20, 3430 Tulln, Austria; michael.sulyok@boku.ac.at; 5 Institut für Chemie, Technische Universität Berlin, Straße des 17. Juni 124, 10623 Berlin, Germany; simon.boecker@chem.tu-berlin.de (S.B.); suessmuth@chem.tu-berlin.de (R.D.S.); 6 Research Cluster “Translational Cancer Therapy Research”, 1090 Vienna, Austria

**Keywords:** cyclohexadepsipeptide, beauvericin, cervix carcinoma, colorectal carcinoma, therapy

## Abstract

Recently, in vitro anti-cancer properties of beauvericin, a fungal metabolite were shown in various cancer cell lines. In this study, we assessed the specificity of this effect by comparing beauvericin cytotoxicity in malignant versus non-malignant cells. Moreover, we tested in vivo anticancer effects of beauvericin by treating BALB/c and CB-17/SCID mice bearing murine CT-26 or human KB-3-1-grafted tumors, respectively. Tumor size and weight were measured and histological sections were evaluated by Ki-67 and H/E staining as well as TdT-mediated-dUTP-nick-end (TUNEL) labeling. Beauvericin levels were determined in various tissues and body fluids by LC-MS/MS. In addition to a more pronounced activity against malignant cells, we detected decreased tumor volumes and weights in beauvericin-treated mice compared to controls in both the allo- and the xenograft model without any adverse effects. No significant differences were detected concerning percentages of proliferating and mitotic cells in tumor sections from treated and untreated mice. However, a significant increase of necrotic areas within whole tumor sections of beauvericin-treated mice was found in both models corresponding to an enhanced number of TUNEL-positive, i.e., apoptotic, cells. Furthermore, moderate beauvericin accumulation was detected in tumor tissues. In conclusion, we suggest beauvericin as a promising novel natural compound for anticancer therapy.

## 1. Introduction

Malignant diseases are a major health concern worldwide [1] being the leading cause of death in most US states [2]. Although various treatment options including surgery, radiotherapy, chemotherapy, immunotherapy, and targeted therapeutics have been established [3], results in terms of progression-free and overall survival in several cancer types are still unsatisfying [4]. The most common limitations to currently-available approaches are severe adverse reactions and the development of multidrug resistance [5,6]. Therefore, there is a need for the development of new agents with novel mechanisms of action for cancer treatment [4]. Compounds isolated from different natural sources are promising candidates for the development of novel anticancer drugs, as shown previously [7]. Recently, naturally-occurring cyclic depsipeptides, which consist of hydroxyl- and amino acids linked by amide and ester bonds became of interest. They are secondary metabolites of bacteria, fungi and plants, or originate from algae, cyanobacteria, or sponges of the marine environment [8]. These compounds are known to exert a broad spectrum of biological effects, such as immunosuppressive, antibiotic, antifungal, as well as anti-inflammatory activities. Importantly, they were also shown to exhibit anticancer effects in different tumor models [9,10,11]. Recently, in vitro anticancer activity of the cyclodepsipeptides enniatins (ENNs) [12,13] and beauvericin (BEA) [14,15,16,17] were reported. In the case of ENNs, we showed high tumor cell specificity indicated by clearly-enhanced cytotoxicity against malignant, as compared to non-malignant, cells [12]. In addition, in vivo synergism of enniatin B with sorafenib, a clinically-approved tyrosine kinase inhibitor was observed in a cervical cancer model [18]. Similar anticancer effects were discussed for the structurally-related beauvericin. In the present pilot study we evaluated the cytotoxicity of beauvericin in normal versus malignant cell lines and assessed the in vivo anticancer activity of beauvericin to estimate its therapeutic potential.

## 2. Results

### 2.1. Beauvericin Exerts Enhanced Cytotoxicity in Malignant as Compared to Non-Malignant Cells

Following previous studies in which beauvericin was suggested as potential anticancer drug [15,16,19] we compared the cytotoxic potency of beauvericin against malignant or non-malignant cells in vitro. At sparse conditions (5–10% cell confluency), beauvericin exerted cytotoxic effects on murine colon carcinoma cells (CT-26) and murine non-malignant fibroblasts (NIH/3T3) at micromolar concentrations (Table 1, Figure S1). While IC_25_ values were similar, IC_50_ levels were 1.7-fold higher and IC_75_ values were even 2.4-fold higher for the non-malignant cell line as compared to the malignant CT-26 cells (Table 1). The difference between malignant and non-malignant cells became even more pronounced when treating cells at higher density (50–60%), representing better the in vivo situation. In the murine models, the IC_50_ value for beauvericin in CT-26 cells remained comparable (1.8 µM), in contrast to the IC_50_ value of the NIH-3T3 cells which rose to 9.4 µM. While at 1 µM no reduction of viability was observed in the non-malignant cells, tumor cell viability was already significantly reduced at 0.5 µM beauvericin at these conditions (Figure S3).

Regarding human cell lines, we treated non-malignant keratinocytes (HaCaT), three cervix cancer cell lines (KB-3-1, ME-180, GH354), and two cell lines originating from colon carcinomas (SW480, SW620), with increasing concentrations of beauvericin (Table 2, Figure S2a,b). At sparse conditions, most malignant cell lines were more sensitive towards beauvericin treatment indicated by lower IC-values as compared to HaCaT cells (Table 2, Figure S2a,b). Furthermore, SW620, the most dedifferentiated cell line used derived from a colon carcinoma metastasis [20], was most susceptible to beauvericin with an IC_50_ of 0.7 µM, i.e., 4.7-fold lower as compared to the non-metastatic colon carcinoma cell line SW480 and 5.6-fold lower than the normal HaCaT cells. When treating the cells at higher density (50–60% cell confluency) the difference between non-malignant and malignant cells was distinctly stronger. The IC_50_ values in KB-3-1 and SW480 cells increased only modestly (4.3 and 3.7 µM, respectively) while the one for non-malignant HaCaT cells rose distinctly to above 10 µM (Figure S3).

To estimate the main mechanism underlying the reduced viability after beauvericin treatment, cell cycle and cell death analyses were performed in KB-3-1 cells. While at subtoxic concentrations a cell cycle arrest in the G0/G1-phase was prevalent (Figure S4a), at concentrations >2 µM induction of apoptotic cell death was clearly detectable (Figure S4b). In parallel, cleavage of poly-(ADP-ribose) polymerase (PARP) and caspase 9 was induced and upregulation of the proapoptotic Bcl-2 family members Bim and Bak was observed, while antiapoptotic Bcl-xL was reduced (Figure S4c).

### 2.2. Beauvericin Treatment Induced Reduced Tumor Volumes and Increased Necrosis in an Allograft Mouse Model

**Figure 1:**
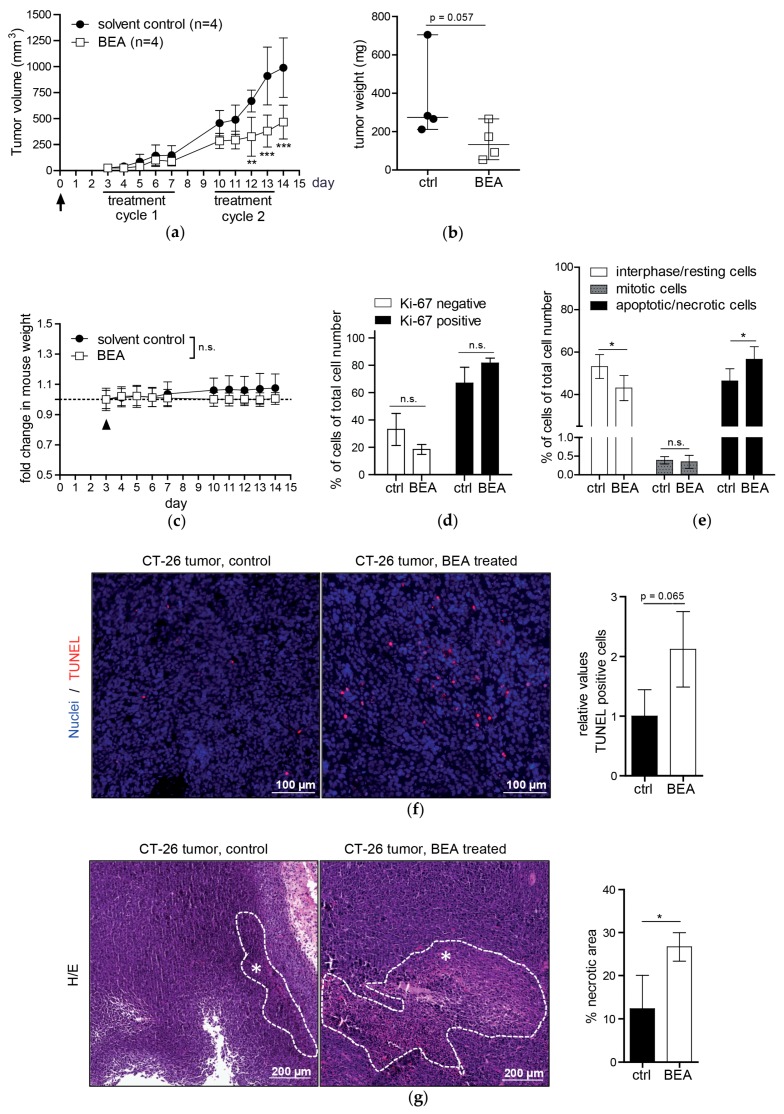
In vivo anticancer activity of beauvericin (BEA) on CT-26-derived tumor allografts. (**a**) On day 0 tumor cells were injected (arrow) and beauvericin was administered in two cycles as indicated. Tumor volumes are given in mm^3^ as mean values (±SD) for the solvent control (black circles) and beauvericin-treated group (open squares); (**b**) After sacrificing all mice on day 14, tumor weights were determined (median tumor weights in mg ± range); (**c**) Body weight of mice was measured during the study on the indicated 10 days and shown as mean fold change (±SD) relativized to baseline levels (dashed line) before treatment start (arrowhead); (**d**) Percentage of Ki-67-negative (open bars) and positive cells (black bars) in tumor sections from four treated and four control mice are shown; (**e**) Interphase and resting cells (open bars), mitotic (gray bars) and apoptotic/necrotic cells (black bars) counted in H/E-stained tumor sections are given in % of total cell number (±SD), counted in at least four optical fields of four tumors of both groups; (**f**) Representative images of tumor sections with TUNEL-positive cells (red) and DAPI-stained nuclei (blue) of a control (left) and of a treated mouse (middle) are shown. Results of TUNEL-positive cells counted in four tumor specimens of both groups respectively are given as relative values compared to the control (right); (**g**) Representative images of H/E-stained tumor sections of a control (left) and of a treated mouse (middle) are shown. Necrotic areas are encircled by white dashed lines and marked by asterisks. Areas of necrotic tissue were quantified by Definiens TissueStudio^®^ 4.0 software from four tumors of both groups, respectively, and are depicted as the percent (±SD) of the total tumor area of the complete section (right). * *p* < 0.05; ** *p* < 0.01; *** *p* < 0.001.

Potential therapeutic effects of beauvericin were investigated in vivo in an allograft mouse model. Three days after subcutaneous injection of murine CT-26 colon-carcinoma cells into the right flank of BALB/c mice tumors were palpable and i.p. treatment with 5 mg beauvericin /kg body weight (bw)/day or with solvent alone was started (Figure 1a). While during the first therapy cycle (days 3–7) only a minor effect on tumor volumes was observed; a marked reduction of tumor growth was detected in all four treated mice during the second treatment cycle (days 10–13). Differences in tumor volumes between the two groups were highly significant (*p* < 0.01) from day 12 onwards (Figure 1a) and culminated in a 52.8% reduction of mean tumor volume in treated mice on day 14 at the end of the study. In addition to tumor volumes, tumor weights were measured showing that three of the four treated mice had lower tumor weights than the control group (Figure 1b). Albeit not statistically significant (*p* = 0.057), an average 60% tumor weight reduction was observed in treated compared to untreated mice in accordance with the significant differences in tumor volumes (Figure 1a).

Throughout the complete study period, mean body weight of mice remained virtually unaltered in both groups and no significant differences were observed between the treatment and the control group until the end of the second treatment cycle (Figure 1c). Furthermore, behavior of the animals was monitored (see materials and methods) yielding no indication for beauvericin-attributed systemic toxicity (data not shown).

To investigate effects of beauvericin on tumor tissues in greater detail histological sections of tumor specimens obtained from treated and untreated mice were stained by different techniques. Staining for the proliferation marker Ki-67—expressed in cells during interphase or M-phase of the cell cycle [21]—revealed a slightly, but not significantly, higher percentage of proliferating tumor cells in the treated compared to the untreated group (81.6 ± 3.7% vs. 66.9 ± 11.7%. Figure 1d). Assessment of fractions of mitotic cells in H/E-stained tumor sections yielded almost identical mean values for both groups (0.4 ± 0.1% control vs. 0.3 ± 0.2% treatment, Figure 1e). However, in the group treated with beauvericin a 22% higher rate of scattered cells with signs of cell death (apoptosis or necrosis) became obvious within viable tumor regions (*p* < 0.05, Figure 1e). In accordance, in TUNEL staining—a sensitive method for the detection of DNA strand breaks during apoptosis [22]—we observed a 2.1-fold increased average apoptosis rate in viable tumor areas of treated versus untreated mice, which did not reach statistical significance (*p* = 0.065, Figure 1f). Additionally, a 2.2-fold increase of necrotic areas was detected in H/E-stained whole tumor sections of beauvericin-treated mice compared to the control group (*p* < 0.05, Figure 1g).

### 2.3. Reduced Growth of Human Tumor Xenografts and Increased Necrosis in Tumor Tissue in Beauvericin-Treated Mice

**Figure 2:**
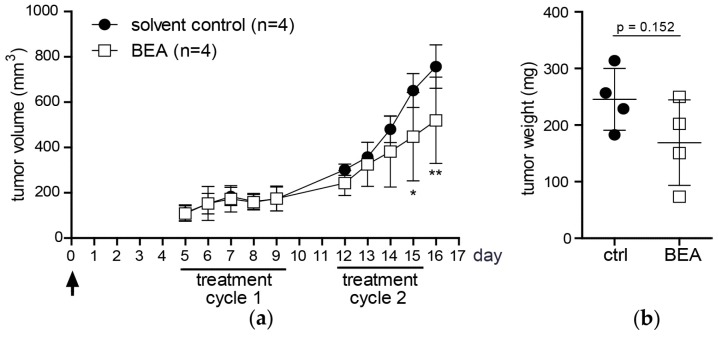

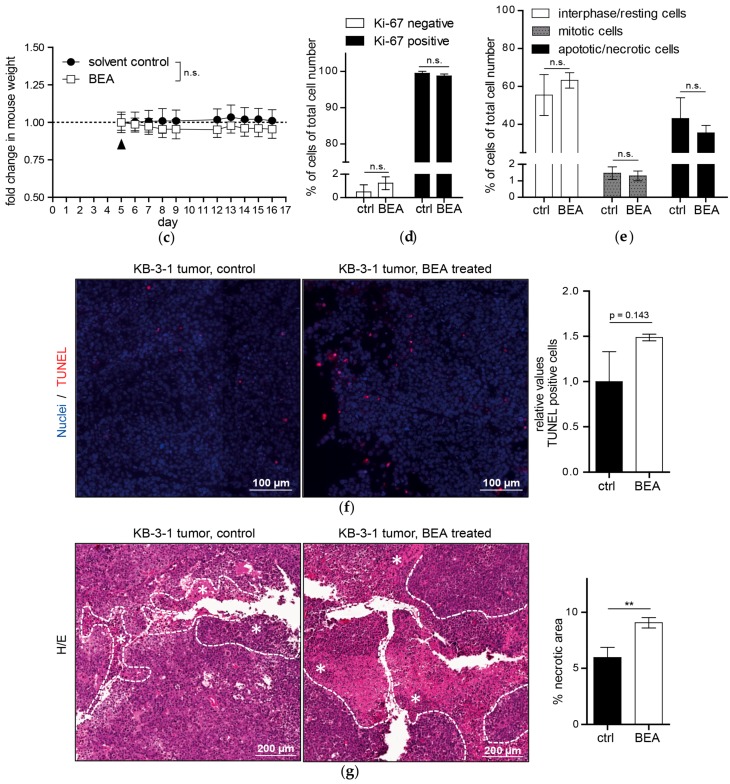
In vivo anticancer activity of beauvericin on KB-3-1-derived tumor xenografts. (**a**) On day 0 tumor cells were injected (arrow) and beauvericin was administered in two cycles as indicated. Tumor volumes are given in mm^3^ as mean values (±SD) for the solvent control (black circles) and beauvericin-treated group (open squares); (**b**) After sacrificing all mice on day 16, tumor weights were determined (mean tumor weights in mg ± SD); (**c**) Body weight of mice was measured during the study on the indicated 10 days and shown as mean fold change (±SD) relativized to baseline levels (dashed line) before treatment start (arrowhead); (**d**) Percentage of Ki-67-negative (open bars) and positive cells (black bars) in tumor sections from four treated and four control mice are shown; (**e**) Interphase and resting cells (open bars), mitotic (gray bars) and apoptotic/necrotic cells (black bars) counted in H/E-stained tumor sections are given in % of total cell number (±SD) and are counted in at least four optical fields of four tumors of both groups; (**f**) Representative images of tumor sections with TUNEL-positive cells (red) and DAPI-stained nuclei (blue) of a control (left) and of a treated mouse (middle) are shown. Results of TUNEL-positive cells counted in four tumor specimens of both groups, respectively, are given as relative values compared to the control (right); (**g**) Representative images of H/E-stained tumor sections of a control (left) and of a treated mouse (middle) are shown. Necrotic areas are encircled by white dashed lines and marked by asterisks. Areas of necrotic tissue were quantified by Definiens TissueStudio^®^ 4.0 software (Definiens^®^, Munich, Germany) from four tumors of both groups, respectively, and are depicted as the percent (±SD) of the total tumor area of the complete section (right). * *p* < 0.05; ** *p* < 0.01.

To study therapy efficacy of beauvericin on human tumor growth, a cervix-carcinoma KB-3-1 xenograft mouse model was used. In accordance with the allograft experiments (Figure 1a) tumor volumes of severe combined immunodeficiency (CB-17/SCID) mice treated with 5 mg/kg bw/day beauvericin were significantly reduced during the second treatment cycle (Figure 2a) with a 31.3% reduction in tumor volume at the end of the experiment (day 16). Likewise, mean tumor weight was lowered by 31.2% in treated as compared to control mice (*p* = 0.152, Figure 2b). In accordance with data of the allograft model (Figure 1c) SCID mice did not show any indications for possible adverse treatment effects. Neither alterations of average body weight (Figure 2c) nor abnormalities in behavior were observed (data not shown).

In Ki-67- and H/E-stained KB-3-1 tumor sections, similar results were obtained as compared to the allograft model in terms of rates of proliferating cells: in the control group 99.5 ± 0.6% and in the treatment group 98.8 ± 0.6% of Ki-67-positive cells were found (Figure 2d) and 1.5 ± 0.4% mitotic cells were counted in the control versus 1.3 ± 0.3% in the treatment group (Figure 2e). While almost no difference in percentages of apoptotic/necrotic cells within viable areas was observed between the two groups in H/E-stained tumor sections (Figure 2e), a 1.5-fold increase of TUNEL-positive cells was detected in tumor specimens of treated mice, indicating an not significant trend (*p* = 0.143) towards an increased rate of tumor cell death under beauvericin treatment (Figure 2f). Similar to the allograft model, in H/E-stained whole tumor sections the proportion of necrotic areas was significantly increased by 34.2% in tumors of beauvericin-treated mice (Figure 2g).

### 2.4. Distribution of Beauvericin in Tissue, Biological Fluids, and Tumor Specimens

**Figure 3:**
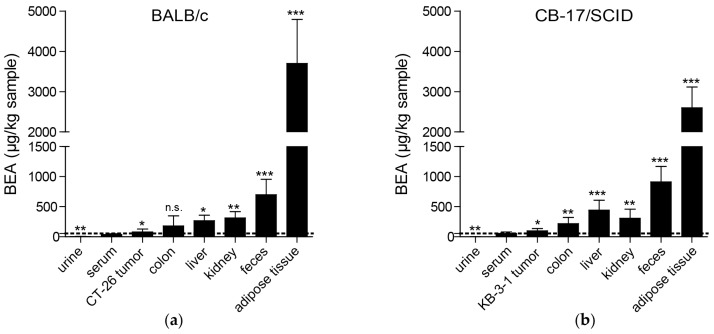
Beauvericin distribution in mouse tissues and biological fluids after 9 days of treatment. Beauvericin levels (µg/kg sample) were determined in duplicates in all tissues indicated. The dashed line indicates the serum levels of beauvericin. Specimens were obtained from each mouse of both, the control (*n* = 4) and the treatment group (*n* = 4) of (**a**) the allograft model or (**b**) of the xenograft tumor model. * *p* < 0.05; ** *p* < 0.01; *** *p* < 0.001.

Previously, a liquid chromatography mass spectrometry (LC-MS/MS) method was established to quantify beauvericin concentrations in various tissues and biological fluids after short-term treatment of healthy mice [19]. In the current study, we investigated tissue distribution of beauvericin after treatment of tumor-bearing mice as outlined above. In the allograft experiment with BALB/c mice beauvericin was measured in urine, serum, feces, and tissues, including CT-26 tumors, colon, liver, kidney, and adipose tissue (Figure 3a), showing a significant 2.3-fold accumulation in tumors (81.0 ± 46.6 µg/kg) compared to serum (35.7 ± 13.5 µg/kg). However, the highest concentrations were found in adipose tissue (3701.3 ± 1091.5 µg/kg, 103.6-fold accumulation compared to serum) followed by feces (698.1 ± 257.2 µg/kg, 19.5-fold), excretory organs (kidney: 312.8 ± 104.6 µg/kg, 8.8-fold; liver: 266.5 ± 91.1 µg/kg, 7.5-fold), and colon (178.5 ± 170.1 µg/kg, 4.9-fold), while beauvericin concentrations in urine samples were very low (3.0 ± 3.3, 0.08-fold). In the xenograft experiment distribution of beauvericin was similar, but showed less pronounced accumulation in KB-3-1 tumor specimens (1.8-fold, 99.9 ± 35.7 µg/kg in tumor vs. 55.7 ± 23.4 µg/kg in serum) and in adipose tissue (2600.5 ± 513.9 µg/kg, 46.7-fold, Figure 3b). Enrichment of beauvericin in colon (217.2 ± 104.0 µg/kg, 3.9-fold), liver (440.9 ± 165.9 µg/kg, 7.9-fold), kidney (305.7 ± 151.0 µg/kg, 5.5-fold), and feces (912.9 ± 256.8 µg/kg, 16.4-fold) was comparable in both models, but in the xenograft experiment levels of beauvericin in urine were below the detection limit.

### 2.5. Concentrations of Aspartate Aminotransferase (AST), Alanine Aminotransferase (ALT), Bilirubin, and Creatinine in Serum Indicate no Liver or Kidney Damage in Beauvericin-Treated CB-17/SCID Mice

**Figure 4:**
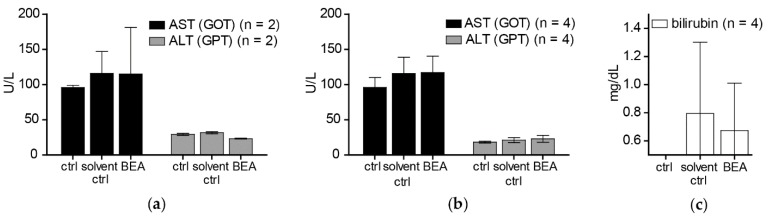
Serum levels of aspartate aminotransferase (AST, black bars) and alanine aminotransferase (ALT, gray bars) were determined in CB17/SCID mice that were untreated (ctrl), treated with solvent (solvent ctrl) or with beauvericin (BEA) (**a**) one day after the last drug application and (**b**) two weeks after therapy finalization; and (**c**) bilirubin concentrations after two weeks of therapy in the sera of untreated (ctrl), solvent treated (solvent ctrl) and beauvericin-treated (BEA) mice are shown. Means (±SD) of replicates are shown for all measurements (**a**–**c**).

Since considerable accumulation of beauvericin in liver and kidney was observed, possible hepato- and nephrotoxic effects of beauvericin were determined by monitoring concentrations of AST, ALT, bilirubin, and creatinine in the sera of beauvericin-treated mice in comparison to solvent-treated and untreated controls. One day after therapy, the serum level of AST in treated mice was 115.3 ± 66.0 U/L, which was similar to that of solvent treated (116.1 ± 30.9 U/L) and untreated (95.9 ± 3.1 U/L) mice (Figure 4a). Likewise, serum levels of ALT, a more specific indicator of liver damage, were 23.3 ± 0.6 U/L in treated mice, which was comparable to the levels determined in the sera of solvent-treated (31.7 ± 1.4 U/L) or untreated (29.4 ± 1.4 U/L) mice (Figure 4a). The levels of total bilirubin, indicative for liver function, and of serum creatinine, reflecting glomerular filtration efficacy, were below the detection limit of 0.5 mg/dL in all mice at the end of the treatment period. As beauvericin was found to clearly accumulate in adipose tissue, a delayed release into the blood could be envisaged. Hence, in addition to measurements immediately after the last drug application, we further quantified these serum parameters two weeks after the last treatment at termination of the experiment. Results did not indicate any significant differences in serum levels of beauvericin-treated (AST: 117.3 ± 23.3 U/L, ALT: 23.1 ± 4.9 U/L, bilirubin: 0.6 ± 0.4 mg/dL), solvent treated (AST: 115.8 ± 23.3 U/L, ALT: 21.2 ± 3.6 U/L, bilirubin: 0.7 ± 0.5 mg/dL) and untreated (AST: 96.2 ± 13.9 U/L, ALT: 18.4 ± 1.4 U/L, bilirubin: <0.5 mg/dL) mice (Figure 4b,c), while serum creatinine was below the detection limit (0.5 mg/dL) in all samples.

## 3. Discussion

This study addressed in vivo anticancer efficacy and the therapeutic window of beauvericin. First, conforming to results from the structurally related enniatins [12] we found that beauvericin exerts modestly stronger cytotoxic effects in some malignant versus non-malignant cell types in vitro. The molecular mechanisms underlying these different sensitivities of cancer cell models are not fully understood but might include ABC-transporter-mediated drug efflux mechanisms or altered activation of cell survival pathways [23,24]. In addition, when seeding non-malignant fibroblasts or keratinocytes, but also cancer cells at higher density to mimic the tissue situation, beauvericin cytotoxicity markedly dropped in non-malignant cell types and the differences to cancer cells became more distinct. This selectivity is important for potential in vivo applications where cell death should be triggered in cancer cells while leaving non-malignant cells and tissues unaffected. In addition, our data revealed higher cytotoxic potency of beauvericin in colon carcinoma SW-620 cells from a metastatic lesion compared to the moderately dedifferentiated primary tumor cell line SW-480 of the same patient [20]. This is in agreement with migration inhibition by sublethal beauvericin concentrations in metastatic cancer cells (PC-3M, prostate cancer; MDA-MB-231, breast cancer) [15]. Together this data indicates that beauvericin might target especially dedifferentiated and invasive cancer types.

Investigating in vivo effects of beauvericin in mouse models of murine and human carcinomas, beauvericin treatment significantly reduced tumor volumes as compared to solvent-treated controls. Likewise, mean tumor weights were lower in the beauvericin-treated group of the xenograft and, even more pronounced, of the allograft model. Even though tumor growth was not completely inhibited, these effects suggest therapeutic potential of beauvericin, which may be further enhanced by optimization of the dose and the treatment schedule.

To examine mechanisms underlying the therapeutic effect of beauvericin histological stainings of tumor specimens were performed. In Ki-67-stained tissues the fractions of proliferating (Ki-67-positive) and resting (G0) cells (Ki-67-negative) did not show significant differences between treated and untreated groups in either model corroborated by similar rates of mitotic cells. Therefore, tumor growth reduction by beauvericin treatment was not caused by altered proliferation rates of malignant cells. Counting apoptotic/necrotic cells in H/E-stained tumor section, we observed a higher percentage of cells exhibiting apoptotic and necrotic features in beauvericin-treated mice, especially in the allograft model. Correspondingly, a distinct increase in TUNEL-positive cells, characteristic of apoptosis, was detected. Albeit, due to high variability between different regions of viable tumor parts, this alteration was not statistically significant. Whether uneven distribution of beauvericin in tumor nodules caused this variability needs to be determined. However, our results confirmed previous studies where beauvericin-induced apoptosis in diverse cancer cell types was shown in vitro [16,17,23,24].

The exact mechanisms of cell death induction by beauvericin is not yet established. However, in cervix carcinoma cells, we detected a G0/G1 phase arrest at subtoxic concentrations of beauvericin, followed by apoptosis induction at higher concentrations which was accompanied by the activation of the intrinsic mitochondrial cell death pathway. Additionally, the cytotoxic effects of beauvericin were discussed to be based on its ionophoric characteristics increasing cytoplasmic calcium concentrations and stimulating calcium-dependent endonucleases finally resulting in DNA fragmentation and apoptosis [25]. Furthermore, others have also shown an influence on mitochondrial membrane potential [26,27], increased cytochrome C release followed by caspase 3 activation [17,27], a boost of reactive oxygen species (ROS) production [26], interaction with NF-KB [28] and/or MAPK pathways [16,28], as well as necrotic cell death [28] were suggested to underlie the multifaceted actions of beauvericin. Several of these suggested modes of action, like the boost of ROS production and the interaction with oncogenic NF-KB and/or MAPK pathways might have distinctly stronger impacts on malignant as compared to non-malignant cells and, hence, might contribute to the observed cancer selectivity. Cancer cells, for example, suffer from enhanced oxidative stress and are vulnerable to ROS-generating compounds [29]. For the MAP kinase pathway, inhibitory compounds are already in clinical use against cancer [30]. However, further investigations are necessary to estimate which cancer types might be primary targets for beauvericin treatment and which biomarkers might help to stratify respective patient subgroups.

Additionally to dispersed apoptotic/necrotic cells in the viable tumor, necrotic tissue areas—especially in the centers of the tumors—were significantly enhanced by beauvericin treatment in both tumor models. This is not necessarily a consequence of necrotic cell death but might be induced by focal, but massive, apoptosis of cancer cells or tissue breakdown due to starvation and lack of oxygen. This would suggest an interference of beauvericin with nutrient and oxygen delivery into malignant tissues probably based on inhibition of angiogenic processes. In accordance with this finding, anti-angiogenic activity of subtoxic beauvericin concentrations on human umbilical vein endothelial cells (HUVEC-2) has been described [15]. Likewise, in our hands both vessel forming and migratory ability of HUVEC cells were distinctly inhibited by subtoxic concentrations of beauvericin (unpublished data). Hence, reduction of blood supply in combination with the cytotoxic activity of beauvericin against tumor cells are likely to underlie the extensive necrosis detected in tumors of beauvericin-treated mice. Therefore, experiments investigating the impact of beauvericin on blood supply of tumors are currently initiated.

In our in vivo studies we observed a more pronounced anticancer activity of beauvericin on tumors in the allograft than in the xenograft model. This might result from the different cell-types used, i.e., cells derived from a colon carcinoma (CT-26) in the allograft and from a cervix carcinoma (KB-3-1) in the xenograft model. Accordingly, the former proved to be more susceptible to beauvericin also in vitro (IC_50_ for CT-26: 1.8 µM, for KB-3-1: 3.1 µM). Alternatively, the stronger activity in the allograft model might indicate a contribution of immune-related factors to the anticancer activity of beauvericin. Several chemotherapeutic agents support activation of tumor-targeting T-cell subclones partly based on enhanced tumor antigen presentation [31]. This immune-stimulating effect—synergizing with direct cytotoxic activity against malignant cells—is definitely lacking in SCID mice without functional B- and T-cells. However, immune-inhibitory effects of beauvericin have also been described in a Crohn’s disease model [32]. Consequently, beauvericin might have an effect on invasion of immune cells e.g., T-cells, into the malignant tissue which is currently addressed in our allograft model. Generally, we did not see any signs of adverse effects or immune-related reactions in beauvericin-treated mice indicated by stable body weight, unaltered general physiological conditions (e.g., activity and coat appearance) or lack of abnormal behavior (e.g., grooming, fatigue). In addition, no signs of tissue alteration or inflammatory responses in H/E stained tissue sections of diverse organs were detected. Thus, we conclude that major adverse effects of beauvericin are unlikely.

As already suggested based on the lipophilicity of beauvericin [25], we found the highest beauvericin concentrations in adipose tissue followed by feces, kidney, and liver tissue. However, we also detected moderate beauvericin accumulation in tumor tissues in both mouse models in comparison to serum levels. In urine samples, we only detected minor beauvericin concentrations implying negligible renal clearance of the compound. Fecal enrichment, however, was also reported [25] suggesting elimination of beauvericin mainly through feces.

In contrast to the cytotoxic activity of beauvericin on non-malignant fibroblasts and keratinocytes shown in our in vitro experiments, no macro- and microstructural tissue changes were observed in liver and kidney [19], despite accumulation of beauvericin in these organs. In line with this observation, no alterations of serum markers indicating tissue damage (AST, ALT) or impairment of kidney or liver function (bilirubin, creatinine) were detected both in treated and in control mice immediately and two weeks after the treatment period. This discrepancy between cytotoxic effects of beauvericin in vitro and lack of obvious adverse reactions in vivo might be explained at least in part by the loss of beauvericin cytotoxicity against non-malignant but not against malignant cell types at higher cell density. Additionally, pharmacokinetic parameters and blood vessel integrity effects might lead to tissue-specific drug exposure alterations in the in vivo situation.

Although in this study we observed a significant enrichment of beauvericin in tumors compared to serum levels, the accumulation was much more pronounced in adipose tissue, liver, kidney, or colon. In general, several approaches are possible to improve such sub-optimal tumor accumulation. Hence, drugs might be chemically modified to obtain derivatives with enhanced pharmacological characteristics. Alternatively, nanoformulations of several anticancer drugs were proven to be superior compared to the native substances also in clinical applications, such as the approved liposomal doxorubicin (Doxil^®^) [33]. Therefore, we plan to develop beauvericin derivatives and/or nanoformulations with improved therapeutic windows.

Beauvericin exerted a more pronounced anticancer activity in single drug regimens as compared to the closely related compound ENN B [18], maybe due to differences in metabolization. While no metabolites of beauvericin could be detected in mice after three days of treatment, ENN B was processed to three phase I metabolites [19]. Similar results were gained in vitro [34]. Due to this obviously higher metabolic stability sustained concentrations of beauvericin may be achieved in vivo. Additionally, rapid acquisition of beauvericin resistance seems unlikely as we did not induce beauvericin-unresponsiveness of KB-3-1 cells during a two-year in vitro selection process [24]. These favorable pharmacokinetic characteristics endorse the therapeutic potential of beauvericin.

## 4. Conclusions

In conclusion, we identified beauvericin as a modestly tumor-selective anticancer agent in vitro, exerting distinct activity and favorable tolerability in vivo in an allo- and a xenograft model of colon- and cervix cancer, respectively. Although tumor enrichment and the therapeutic margin of beauvericin still needs to be improved, our observations suggest further preclinical development of this natural compound as anticancer agent.

## 5. Materials and Methods

### 5.1. Chemicals

Beauvericin was purchased from BioAustralis (Smithfield, Australia) and, for animal experiments, purified from *Beauveria bassiana* (ATCC 7159). The culture conditions were adopted from Xu et al. [14] and the biomass harvested by suction filtration. The mycelium was lyophilized and extracted with ethyl acetate. The solvent was evaporated and the brownish residue resolved in methanol. Insoluble residues were removed by filtration and the solvent evaporated. The residues were dissolved in acetonitrile/water (80:20 *v/v*) and the solution was centrifuged to remove insoluble particles. The supernatant was then subjected to reversed phase chromatography using a GROM-Sil 120 ODS-5 HE, 10 µm, 250 × 20 mm column (Grace GmbH and Co KG, Worms, Germany) on an Agilent 1100 series preparative HPLC system (Agilent Technologies, Waldbronn, Germany) running isocratically on acetonitrile (+0.1% formic acid)/water (+0.1% formic acid) (70:30 *v/v*) with a flow rate of 15 mL/min. Beauvericin containing fractions were pooled, acetonitrile was evaporated and water was removed by lyophilization. Purity of the compound was verified by LC-MS on an Agilent ESI-Triple-Quadrupol-MS, 6460 Series (Agilent Technologies, Waldbronn, Germany) and by nuclear magnetic resonance (NMR) on a Bruker Avance III 700 MHz-NMR spectrometer (Bruker, Karlsruhe, Germany). Stock solutions of beauvericin were prepared in DMSO and stored at −20 °C.

### 5.2. Cell Culture

All cancer cell lines used for this study are described in Table S1. Cultures were regularly screened for *Mycoplasma* contamination.

### 5.3. Cell Viability Assay

For the cell lines KB-3-1 and SW480 2 × 10^3^ cells, for NIH/3T3, CT-26, HaCaT, and GH354 cells 3 × 10^3^ cells and for ME-180 and SW620 cells 4 × 10^3^ cells were seeded into 96-well pates and incubated at 37 °C (5% CO_2_) overnight. All cell numbers corresponded to a cell monolayer confluency of 5–10% 24 h after seeding and immediately before beauvericin treatment. For higher cell confluency, NIH/3T3, CT-26, HaCaT, KB-3-1, and SW480 cells were grown to a cell density of approximately 50–60% before treatment. Cells were exposed to increasing concentrations of beauvericin for 72 h. The percentage of viable cells was detected after incubation with 3-(4,5-dimethylthiazol-2-yl)-2,5-diphenyltetrazolium bromide (MTT) at 37 °C for 1–4 h, depending on the cell line, according to the user manual (EZ4U, Biomedica, Vienna, Austria). Cell viability after 72 h was determined and concentrations of beauvericin leading to a reduction of cell number by 25%, 50%, and 75% (IC_25_, IC_50_, IC_75_), respectively, were calculated from whole dose-response curves. All experiments were conducted using full-growth media with 10% FBS in triplicate and repeated three times. The cell confluency was analyzed by Image J 1.50i (NIH, New York, NY, USA).

### 5.4. In Vivo Allo- and Xenograft Experiments

For the allograft experiment, 4 × 10^5^ CT-26 cells were resuspended in 50 µL RPMI medium and injected subcutaneously into the right flank of eight, 6–8 weeks old, male BALB/c mice that were obtained from Harlan Laboratories (San Pietro al Natisone, Italy). Likewise, in the xenograft experiment, 1 × 10^6^ KB-3-1 cells in 50 µL RPMI medium were injected subcutaneously into the right flank of eight, 6–8 weeks old, male CB-17/IcrHanHsd-Prkdc severe combined immunodeficiency (SCID) mice. Of each group, four animals were randomly assigned to the control or to the treatment group. After the tumor was palpable and reached an approximate size of 25 mm^3^ the respective mouse was either treated intraperitoneally with 5 mg/kg bw/day beauvericin (dissolved in 10% DMSO) as described previously [19] or, for the control group, with solvent alone (10% DMSO). All animals were kept under pathogen-free conditions and all procedures were performed in a laminar flow hood. Effects of the treatment were assessed by daily recording of tumor size with a microcaliper and parameters indicating the animals’ overall health condition (e.g., body weight, fatigue, grooming, ragged coat, food and fluid consumption). Tumor volumes (mm^3^) were calculated using the formula: (length × width^2^)/2. After two therapy cycles of four to five days, and 24 h after the last beauvericin injection, animals were sacrificed by cervical dislocation after anesthesia (Ketavet^®^/Rompun^®^ mix) to collect blood by heart puncture. The tumor mass was weighed and organs and tissues for immunohistochemical experiments were fixed in 4% formalin/PBS (Roti^®^-Histofix 4%, Roth, Karlsruhe, Germany) or shock-frozen in liquid nitrogen and stored at −80 °C until the samples were prepared for LC-MS/MS analysis. The experiments were approved by the ethics committee of the Austrian Federal Ministry of Science, Research, and Economy (BMWF-66.009/0084-II/3b/2013, date of approval: 5 September 2013) and performed in line with the Arrive guidelines for animal care and protection and with guidelines from the Austrian Animal Science Association and from the Federation of European Laboratory Animal Science Associations (FELASA).

### 5.5. Immunohistochemistry

From each mouse from both the control (*n* = 4) and the treatment group (*n* = 4), tissue samples were formalin-fixed, paraffin-embedded and used to prepare serial 3 µm sections. Then, slices were deparaffinized and rehydrated. To evaluate the percentages of interphase/resting, mitotic and dead (apoptotic/necrotic) cell fractions in tumor specimens, sections were stained with hematoxylin and eosin (H/E) by means of standard protocols. Numbers of the three cell fractions were analyzed in a blinded setup in at least four images of H/E-stained tumor sections, taken under a 40 × objective microscope lens. In addition, for the quantification of areas of dead tumor tissue in whole tumor sections, the H/E-stained slides were scanned with a Pannoramic MIDI automated slide scanner (3DHISTECH, Budapest, Hungary) and evaluated using Definiens’ TissueStudio^®^ 4.0 (Definiens^®^, Munich, Germany) software and Pannoramic Viewer (3DHISTECH) software. To evaluate the fraction of apoptotic cells in tumor sections a terminal deoxynucleotidyl transferase (TdT) deoxyuridine triphosphate (dUTP) nick end labeling (TUNEL) assay was performed applying the In Situ Cell Death Kit TMR (red) (Roche Diagnostics, Mannheim, Germany) according to the manufacturer’s protocol. Afterwards, slides were covered with Vectashield anti-fade mounting medium with DAPI (H-1200, Vector Laboratories, Burlingame, CA, USA). Slides were protected from light until they were scanned with a Pannoramic MIDI automated slide scanner (3DHISTECH) and analyzed via digital image analysis software (Definiens’ TissueStudio^®^ 4.0, Definiens^®^). In order to visualize the proliferative cell fraction (cells in the interphase or M-phase) of tumors, KB-3-1 tumor sections were stained with the Ki-67 (clone MiB-1) antibody from DAKO (1:100; Glostrup, Denmark) and for CT-26 tumor sections with Ki-67 (1:100; D3B5, rabbit, Cell Signaling, Danvers, USA). The primary antibody was incubated for 30 min at room temperature in a humid chamber and after rinsing the slides for 3 min in PBS + 0.1% Tween, the UltraVision LP detection system was applied according to the manufacturer’s instructions (Thermo Fisher Scientific, Massachusetts, MA, USA). Sections were counterstained with hematoxylin. For analyzing the percentages of Ki-67-negative and -positive cells in the tumor sections, slides were scanned and analyzed via digital image analysis software (Definiens’ TissueStudio^®^ 4.0, Definiens^®^).

### 5.6. LC-MS/MS Analysis

Concentrations of beauvericin in tissue samples were analyzed according to Rodríguez-Carrasco et al. [19]. Briefly, tissues were collected from each mouse of both the control (*n* = 4) and the treatment group (*n* = 4). Samples of which were thawed on ice, divided and weighted. Afterwards, each slice was placed into a Precellys^®^ hard tissue homogenizing CK28 tube (VWR, Radnor, PA, USA). After adding 2 mL of acetonitrile (LC-MS LiChrosolv^®^, Merck Millipore, Darmstadt, Germany) to each tube, samples were centrifuged for four cycles with 6000 rpm for 30 s with a 30 s break. Beauvericin from blood and urine samples was extracted by mixing 50 µL of sample with 1.5 mL acetonitrile and vortexing for 15 s. The supernatant was transferred into a fresh tube and stored at −80 °C until the LC-MS/MS analysis was performed. Therefore, a QTrap 5500MS/MS system (Applied Biosystems, Foster City, CA, USA) coupled to a TurboV electrospray ionization (ESI) source and a 1290 series UHPLC system (Agilent Technologies, Waldbronn, Germany) were used. Chromatographic separation was achieved at 25 °C on a Gemini^®^ C18 column (150 × 4.6 mm i.d., 5 µm particle size) connected to a C18 security guard cartridge (4 × 3 mm i.d.; all from Phenomenex, Torrance, CA, USA) with a flow rate of 1 mL/min. Elution was performed in binary gradient mode and both mobile phases contained 5 mM ammonium acetate and were composed of methanol/water/acetic acid 10:89:1 (*v*/*v*/*v*; eluent A) and 97:2:1 (*v*/*v*/*v*; eluent B), respectively. In the first 2 min of elution 100% eluent A was used, afterwards the proportion of eluent B was increased linearly to 50% within 3 min followed by a linear increase of B to 100% within 9 min. Finally, after a hold-time of 4 min with 100% of eluent B, the column was re-equilibrated with 100% eluent A for 2.5 min. ESI-MS/MS was performed in the scheduled selected reaction monitoring (sSRM) mode in positive mode and the target scan time was set to 1 s. The ESI source was set as follows: source temperature 550 °C, curtain gas 30 psi (206.8 kPa of max. 99.5% nitrogen), ion source gas 1 (sheath gas) 80 psi (551.6 kPa of nitrogen), ion source gas 2 (drying gas) 80 psi (551.6 kPa of nitrogen), ion-spray voltage + 5500, collision gas (nitrogen) medium.

### 5.7. Quantitative Determination of Aspartate Aminotransferase (AST), Alanine Aminotransferase (ALT), Bilirubin, and Serum Creatinine

For the measurement of AST, ALT, bilirubin, and creatinine, ten male CB-17/IcrHanHsd-Prkdcscid mice (Harlan Laboratories, San Pietro al Natisone, Italy) that were 6–8 weeks old, were randomly assigned to three groups: two to the control (i.e., no treatment), four to the solvent control (10% DMSO), and four to the beauvericin treatment group (5 mg/kg bw/day beauvericin). Mice were treated in two cycles of five and four days, respectively. One day and two weeks after the last treatment, blood was collected from mice. Samples were allowed to coagulate at room temperature and serum was obtained by two centrifugation steps (1000 rpm, 10 min, 4 °C). Concentrations of AST, ALT, bilirubin, and serum creatinine were determined in serum samples by Reflotron^®^ Plus System (Roche, Basel, Switzerland) according to the manufacturer’s instructions.

### 5.8. Statistics

Data were analyzed using GraphPadPrism 5 software (GraphPad Software Inc., La Jolla, CA, USA). Results are given as mean ± standard deviation (SD), if not indicated otherwise. Tumor volumes of the two groups were compared for each day by two-way ANOVA followed by Bonferroni post-test. For all other statistical analyses an unpaired two-tailed Student’s *t*-test or, for non-parametric data-distribution, a Mann-Whitney test was performed.

## Figures and Tables

**Table 1:** Cytotoxic activity of beauvericin (BEA) in murine cell lines.

Cell Line	Tissue/	BEA (µM)	BEA (µM)	BEA (µM)
	Cell Type	Mean IC_25_^1^ ± SD	Mean IC_50_^1^ ± SD	Mean IC_75_^1^ ± SD
NIH/3T3	embryonic fibroblasts	1.2 ± 0.6	3.1 ± 0.2	6.5 ± 0.7
CT-26	colon carcinoma	1.4 ± 0.2	1.8 ± 0.2	2.7 ± 0.5

^1^ IC_25_, IC_50_, and IC_75_ values were calculated from dose-response curves and are given in means ± SD from at least three independent experiments performed in triplicate.

**Table 2:** Anticancer activity of BEA in human cell lines.

Cell Line	Tissue/	BEA (µM)	BEA (µM)	BEA (µM)
	Cell Type	Mean IC_25_^1^ ± SD	Mean IC_50_^1^ ± SD	Mean IC_75_^1^ ± SD
HaCaT	Keratinocytes	2.7 ± 0.2	3.9 ± 0.4	4.8 ± 0.7
KB-3-1	Cervix carcinoma	2.6 ± 0.9	3.1 ± 0.7	3.6 ± 0.9
ME-180	Cervix metastasis	1.6 ± 0.8	2.2 ± 0.7	4.5 ± 1.2
GH354	Cervix adenocarcinoma	2.2 ± 0.6	3.6 ± 1.2	6.3 ± 2.1
SW480	Colorectal adenocarcinoma	2.1 ± 1.3	3.3 ± 0.3	4.2 ± 2.7
SW620	Colon metastasis (from SW480)	0.3 ± 0.02	0.7 ± 0.1	1.9 ± 0.2

^1^ IC_25_, IC_50_, and IC_75_ values were calculated from dose-response curves and are given in means ± SD from at least three independent experiments performed in triplicate.

## Supplementary Materials

The following are available online at www.mdpi.com/2072-6651/9/9/258/s1, Figure S1: Cell viability of murine fibroblasts NIH-3T3 and colon carcinoma CT-26 cells after treatment for 72 h with the indicated concentrations of beauvericin, Figure S2: Cell viability of beauvericin-treated human malignant versus non-malignant cells, Figure S3: Impact of beauvericin on cell viability of murine non-malignant fibroblasts NIH-3T3 and human non-malignant keratinocytes HaCaT, murine colon carcinoma CT-26, human cervix carcinoma KB-3-1 and human colon carcinoma SW480 cells at higher density, Figure S4: Effects of beauvericin treatment on KB-3-1 cells, Table S1: Description of cell lines used in this study.
