# Supplementary material for: The Natural Fungal Metabolite Beauvericin Exerts Anticancer Activity In Vivo: A Pre-Clinical Pilot Study
Source: Toxins (Basel). 2017 Aug 24;9(9):258. doi: 10.3390/toxins9090258 (PMC5618191; doi:10.3390/toxins9090258)
Supplement: Supplementary file 1 [file toxins-09-00258-s001.pdf]

Supplementary Materials: The Natural Fungal Metabolite Beauvericin Exerts Anticancer Activity In Vivo: A Pre-Clinical Pilot Study

Daniela Heilos, Yelko Rodríguez-Carrasco, Bernhard Englinger, Gerald Timelthaler, Sushilla van Schoonhoven, Michael Sulyok, Simon Boecker, Roderich D. Süssmuth, Petra Heffeter, Rosa Lemmens-Gruber, Rita Dornetshuber-Fleiss and Walter Berger

1. Supplementary Materials and Methods

1.1 Cell Cycle Analysis

KB-3-1 cells (3.5x10^5/per well) were seeded into 6-well plates and treated the next day with the indicated concentrations of beauvericin. After incubation at 37°C for another 24 h, cells were trypsinized, centrifuged (8 min, 300g, RT) and fixed in 70% ethanol and stored at -20°C overnight. Fixed cells were centrifuged (1 min, 6000g) and resuspended in 100 µL of 0.9% NaCl. RNA was digested with 0.2 mg/mL of RNase for 30 min at 37°C. DNA was stained with propidium iodide (PI, 0.01 mg/mL, Sigma-Aldrich St. Louis, Missouri, USA) for 30 min at 4°C in the dark. Afterwards, cells were analyzed by flow cytometry using FACS Calibur (BD Biosciences, Franklin Lakes, New Jersey, USA) as described previously [1]. CellQuest Pro software (BD Biosciences) was utilized to determine percentages of the respective cell cycle phases.

1.2 Annexin-V/PI Staining

KB-3-1 cells were seeded (2x10^{5}cells/well) and treated on the following day for 24 h with the indicated concentrations of beauvericin. Cells were trypsinized, centrifuged (300g, 5 min) and resuspended in 100 µL buffer A (10 mM HEPES, 140 mM NaCl and 2.5 mM $CaCl_2$) and stained for 15 min in the dark with PI (1 µg/mL) and Annexin-V-APC (20 µL/mL, BD Biosciences). Two-hundred µL of buffer A were then added and 10,000 cells were measured by FACS analysis (FACSCalibur; Becton Dickinson). Percentages of viable, early and late apoptotic cells and of necrotic cells were determined by CellQuest Pro software (Becton, Dickinson and Company, NY, USA).

1.3 Western Blot Analysis

Total protein lysates of KB-3-1 cells after 24 h-treatment with beauvericin were prepared according to standard procedures, separated by SDS-PAGE, and transferred to a polyvinylidene difluoride membrane for Western blotting as described previously [1]. The following primary antibodies specific for the following targets were diluted 1:1000 in TBST/3% BSA (w/v): PARP, Bak, Bax, Bim, Bcl-xL (polyclonal, Cell Signaling Technology, Beverly, MA, USA), caspase 9, cl. caspase 9 (monoclonal, Cell Signaling Technology) and β-actin (monoclonal, Clone AC-15, Sigma-Aldrich). Secondary horseradish peroxidase-labeled antibodies (goat anti-rabbit or anti-mouse, Santa Cruz Biotechnology, Dallas, Texas, TX, USA) were diluted 1:10,000 in TBST/1% BSA.

2. Supplementary Figures

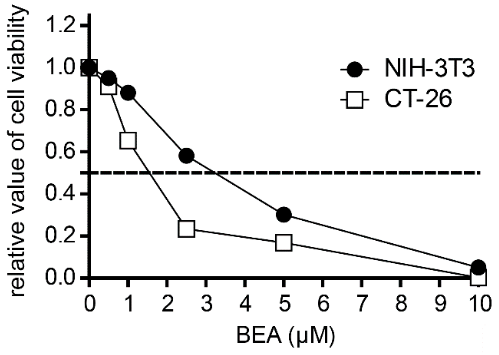

Figure S1. Cell viability of murine fibroblasts NIH-3T3 (black circles) and colon carcinoma CT-26 cells (open squares) after treatment for 72 h with the indicated concentrations of beauvericin (BEA) is shown. For each cell line, viability was compared to untreated cells. Mean relative values (± SD) of triplicate of one representative experiment out of three are shown. The IC_{50} level is indicated by the dashed line.

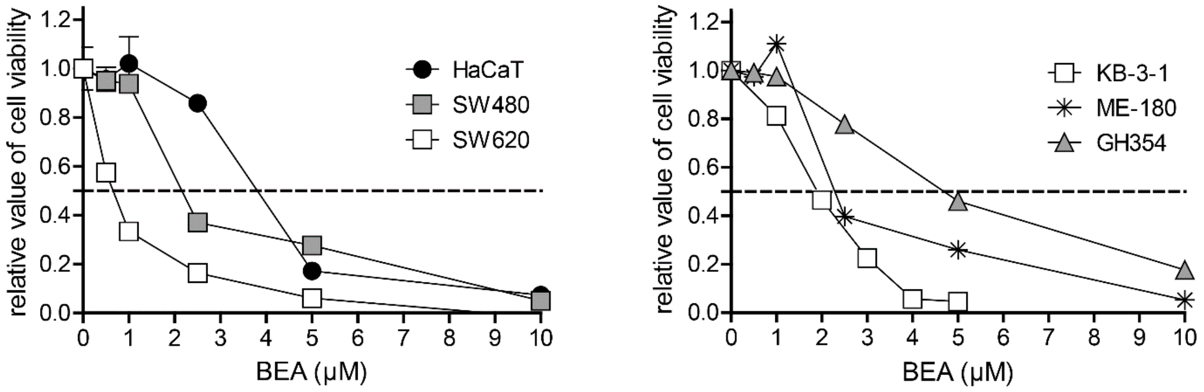

(**a**) (**b**)

Figure S2. Cell viability of beauvericin-treated human malignant versus non-malignant cells. (a) Non-malignant human keratinocytes (HaCaT, black circles) and colon carcinoma cell lines SW480 (gray squares) and SW620 (open squares) as well as (**b**) cervix carcinoma cell lines KB-3-1 (open squares), ME-180 (black asterisk) and GH354 (gray triangle) were treated with the indicated concentrations of beauvericin for 72 h. For each cell line, viability was compared to untreated cells. Mean values (± SD) of triplicate of one representative experiment are given. The IC_{50} level is marked with a dashed line.

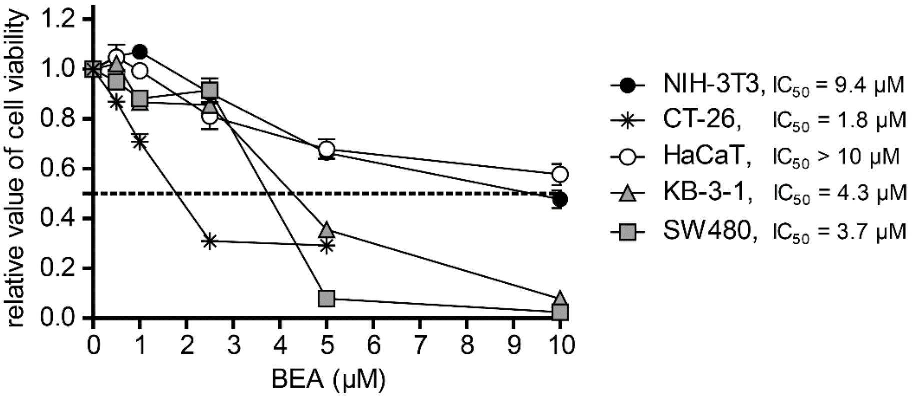

Figure S3. Impact of beauvericin (BEA) on cell viability of murine non-malignant fibroblasts NIH-3T3 (black circles), human non-malignant keratinocytes HaCaT (open circles), murine colon carcinoma CT-26 (asterisks), human cervix carcinoma KB-3-1 (gray triangle) and human colon carcinoma SW480 cells (gray square) at higher density. Cells were grown to a 50-60% confluence and treated with the indicated concentrations of beauvericin for 72 h. The IC_{50} level is indicated by the dotted line. Mean values (± SD) of one representative experiment in triplicate are shown.

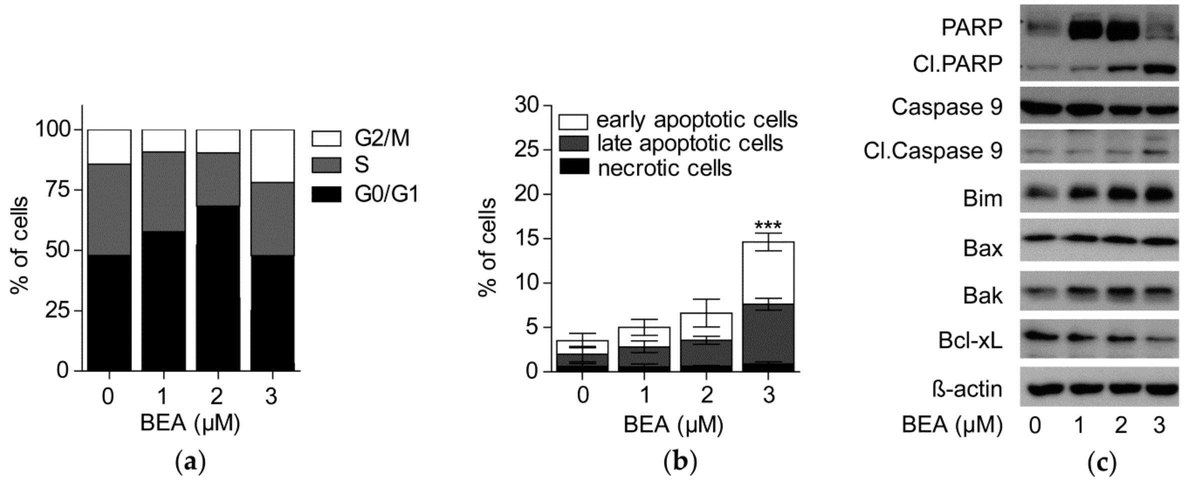

Figure S4. Effects of beauvericin treatment on KB-3-1 cells. (**a**) Percentages of cells in the G2/M (open bars), S (gray bars) or G0/G1 (black bars) phase of the cell cycle among viable cells (=100%) were determined by PI staining after treatment for 48 h with the indicated concentrations of beauvericin. (**b**) Mean percentages (± SD) of early (white bars) and late apoptotic (gray bars) and of necrotic cells (black bars) were determined by Annexin-V/PI staining after cells were treated for 24 h with the indicated concentrations of beauvericin. Significant differences to untreated controls are marked by asterisks (***$p<0.001$). (**c**) Expression of PARP and caspase 9 and amount of cleaved (Cl.) PARP and cleaved Caspase 9, as well as protein expression of Bim, Bax, Bak and Bcl-xL in beauvericin-treated (24 h) and untreated cells were determined by Western Blotting. β-actin served as loading control.

3. Supplementary Table

Table S1. Description of cell lines used in this study.

Cell line (ATCC Nr.)	Tissue (organism)	Medium	Source
3T3/NIH (CRL-1658)	fibroblasts (mouse)	DMEM + 10% FBS	ATCC
CT-26 (CRL-2638)	colon carcinoma-derived cell line (mouse)	DMEM/Ham's Nutrient Mixture F12 (Sigma Aldrich, St. Louis, USA) + 10% FBS	ATCC
GH354 (CRL-13003)	cervix adenocarcinoma- derived cell line (human)	RPMI 1640 + 10% FBS	ATCC
HaCaT	keratinocytes (human) [2]	DMEM + 10% FBS	Dr. N.E. Fusenig (Heidelberg, Germany)
KB-3-1	HeLa derivative, cervix carcinoma-derived cell line (human)	RPMI 1640 + 10% FBS	Dr. Shen (Bethesda, USA)
ME-180 (HTB-33)	cervix; derived from metastatic site: omentum (human)	RPMI 1640 + 10% FBS	ATCC
SW480 (CCL-228)	colorectal adenocarcinoma, Dukes type B-derived cell line (human)	MEME + 10% FBS	ATCC
SW620 (CCL-227)	colon; cell line derived from metastatic site: lymph node (human)	MEME + 10% FBS	ATCC

ATCC, American Type Culture Collection, Rockville, MD, USA; DMEM, Dulbecco's modified Eagle's medium; RPMI, Roswell Park Memorial Institute; MEME, Minimum Essential Medium Eagle medium; FBS, fetal bovine serum (Sigma-Aldrich, St. Louis, USA);

References

1. Dornetshuber-Fleiss, R.; Heffeter, P.; Mohr, T.; Hazemi, P.; Kryeziu, K.; Seger, C.; Berger, W.; Lemmens-Gruber, R. Destruxins: Fungal-derived cyclohexadepsipeptides with multifaceted anticancer and antiangiogenic activities. *Biochem. Pharmacol.* **2013**, 86, 361–377.
2. Boukamp, P.; Petrussevska, R.T.; Breitkreutz, D.; Hornung, J.; Markham, A.; Fusenig, N.E. Normal keratinization in a spontaneously immortalized aneuploid human keratinocyte cell line. *J. Cell Biol.* **1988**, *106*, 761-771.
